# Leveraging antimicrobial stewardship programs in response to the coronavirus disease 2019 (COVID-19) public health emergency

**DOI:** 10.1017/ash.2022.34

**Published:** 2022-03-14

**Authors:** Torsten A. Joerger, Laura L. Bio, Lauren M. Puckett, Hayden T. Schwenk

**Affiliations:** 1 Division of Infectious Diseases, Department of Pediatrics, Stanford University School of Medicine, Stanford, California; 2 Department of Pharmacy, Lucile Packard Children’s Hospital Stanford, Palo Alto, California

## Abstract

The coronavirus disease 2019 (COVID-19) pandemic has strained antimicrobial stewardship programs (ASPs) but offered new opportunities. This review summarizes the impact of the COVID-19 pandemic on ASPs, review the contributions ASPs have made in the pandemic response, and highlight the potential role of ASPs in future pandemics.

Antimicrobial stewardship programs (ASPs) work to optimize the use of antimicrobials to improve clinical outcomes, decrease adverse effects of antimicrobials, and lower costs. After implementation of The Joint Commission standard requiring all hospitals to have an ASP in 2017, these programs are now ubiquitous, with 85% of hospitals meeting all of the Centers for Disease Control and Prevention (CDC) recommended ASP core elements in 2018.^1,2^ ASPs have been shown to decrease length of stay, antibiotic duration, antibiotic expenditures, rates of *Clostridioides difficile* infection, and rates of antimicrobial resistance.^3–6^ The coronavirus disease 2019 (COVID-19) pandemic has created unprecedented challenges to the healthcare system, particularly ASPs. To highlight this interconnected relationship, we review the historic role of ASPs as part of pandemic response efforts, how ASPs can and have been leveraged to support the pandemic response, the impact of the COVID-19 pandemic on traditional ASP strategies, and how ASPs can ensure their inclusion as part of future pandemic responses.

## Historical perspective

Ahmad et al^7^ demonstrated the role ASPs can have in pandemic response during the H1N1 influenza pandemic when their ASP helped create guidelines for the testing, triaging, and treatment of suspected influenza patients. Nevertheless, ASPs do not appear to have been systematically included in pandemic response efforts before the rise of COVID-19. Indeed, the 2017 Society for Healthcare Epidemiology of America Guidance on Outbreak Response and Incident Management does not mention how ASPs can contribute to an outbreak response.^8^ In the absence of existing standards or historical experience, most ASPs were either indirectly or not at all involved in the early phases of the COVID-19 pandemic response.^9^ Factors that contributed to this blind spot may have included the relatively recent development of antimicrobial stewardship, rarity of global pandemics, limited relationships between ASPs and previously identified pandemic response stakeholders (eg, infection prevention and control [IPC]), and limited ASP visibility within individual hospitals and healthcare systems. However, as the pandemic progressed, and particularly as new and repurposed drugs for the prevention and treatment of COVID-19 became available, the potential value of ASPs as part of the pandemic response became increasingly apparent.

## ASP attributes relevant to the pandemic response

The beginning of the COVID-19 pandemic was marked by tremendous uncertainty, characterized by a lack of information about an emerging pathogen, absence of proven treatment options, shifts in hospital resources and patient volumes, and significant pressures on hospital finances. Health systems rapidly pivoted by increasing critical care volume to meet demand, canceling elective surgeries, shifting personnel, and moving patient visits to telehealth when possible.^10–13^ To confront these pandemic-related changes, health systems relied on all available resources, including ASPs, which had several features that made them excellent partners for this work.

First, ASPs are increasingly common. ASPs are now required across hospital settings as a condition of participation for the Centers for Medicare and Medicaid Services and for accreditation by The Joint Commission. Therefore, there was a critical mass of stewards that could be leveraged when the pandemic arose. Second, because most members of ASPs have subspecialty training or experience in infectious diseases (ID), they have content expertise that can be utilized as part of the response to emerging pathogens. Third, ASPs have unique skill sets, including prescription audit and feedback, guideline implementation, medication supply management, and investigational drug procurement, that can help support the work of overburdened frontline providers. Fourth, as the pandemic progressed, the importance of clear and consistent messaging about what was and was not known about COVID-19 became increasingly evident. ASPs have historically leveraged behavioral psychology and communication skills to assist stewardship efforts and can be used to facilitate critical messaging related to the rapidly changing knowledge about COVID-19. Lastly, because their work cuts across entire healthcare systems, ASPs are likely to have diverse personal and professional networks.

These relationships are also likely to include teams that are critically important to pandemic response, including the microbiology/virology laboratory and IPC. Furthermore, because the stewardship community is relatively small, there are interinstitutional relationships that facilitate collaboration and learning based on the experience at other centers.

## Impact of ASPs on the COVID-19 pandemic

ASPs have made many important contributions to the COVID-19 pandemic response, including the management and mitigation of supply shortages. At the start of the pandemic, COVID-19 laboratory tests were not widely available and frequently had a long turnaround time.^14^ Shortages of swabs used for respiratory pathogen panels and *Staphylococcus aureus* PCRs also occurred, so ASP teams encouraged use of culture-based methods of detection to ensure appropriate work up of infections continued to occur.^15^ Personal protective equipment (PPE) was also in short supply; healthcare workers needing to don new PPE every time they entered a patient’s room. ASPs worked to reduce healthcare worker room entries and PPE needs by consolidating and adjusting timing of medication administrations, converting therapy from IV to PO to decrease length of stay, and decreasing unnecessary vancomycin monitoring.^16–19^

Electronic health records (EHR) have been increasingly leveraged by ASP teams to support stewardship activities and the COVID-19 pandemic has seen a number of ASPs use them for pandemic response.^20^ Kubin et al^15^ created a COVID-19–specific alert in the EHR to notify their ASP team when a patient was severe acute respiratory syndrome coronavirus 2 (SARS-CoV-2) PCR positive and when they were on specific medications, such as hydroxychloroquine. This alert system allowed the ASP team to improve patient safety by identifying patients on hydroxychloroquine who had prolonged QT intervals and patients receiving remdesivir without appropriate laboratory monitoring (eg, liver function).^15^ Similarly, 2 other ASPs utilized flags and other EHR tools to identify which COVID-19 patients would most benefit from ID consultation, a resource that became increasingly strained as hospital censuses rapidly increased.^21,22^

The severe morbidity and mortality seen in COVID-19 patients prompted an unprecedented use of antimicrobial agents, both within and outside clinical trials. Given the initial limited supply of medications thought to show antiviral activity against SARS-CoV-2 and uncertainty around which patients would benefit the most from their use, ASP teams applied their expertise to the development of COVID-19 therapeutic guidelines, and they helped allocate these limited resources.^16,23–25^

For example, our own ASP created guidelines for anti–COVID-19 monoclonal antibody treatment at a time when our hospital was only receiving a handful of doses each week. These guidelines helped ensure that these treatments went to the patients who would most benefit as opposed to those that had the easiest access. In addition to COVID-19–specific therapeutics, ASPs worked to mitigate against the surge in antibiotic prescribing in COVID-19 patients that occurred despite evidence that bacterial coinfection and secondary infections were rare.^26–28^

Another challenge of the COVID-19 pandemic response was collating and disseminating the rapidly accumulating knowledge about SARS-CoV-2 and the care of COVID-19 patients. Pierce et al^29^ utilized a smartphone application that had been developed as a repository for institution-specific antimicrobial guidelines to disseminate COVID-19–related content to their medical center. At the time of their publication, these guidelines were downloaded by >3,000 people and individual pages were accessed >200,000 times. Most impressively, the information on the smartphone application was updated >90 times to keep providers abreast of the most current information.^29^ Although Pierce et al^29^ were unable to quantify the clinical impact of this intervention, the work highlights how existing ASP tools can be repurposed as part of the pandemic response.

The challenges of the COVID-19 pandemic have led to increased collaboration, best exemplified by the partnership between ASPs and IPC programs. The rationale for ASP and IPC collaboration, including shared infrastructure, related need for senior hospital leadership support, similar multidisciplinary team structure, shared need for information technology (IT) support, and linked outcome metrics, have been well summarized by Assi et al.^30^ One of the first opportunities for ASP involvement in IPC efforts was early case identification of COVID-19 patients.^25^ Both ASPs and IPCs rely on data collection with help from IT departments; the pandemic highlighted an opportunity for collaboration in acquiring and analyzing these data.^25^ ASPs and IPCs were able to work together on COVID-19 guidelines, ensuring consistent messaging during an evolving pandemic.^30^ Notably, opportunities for ASP and IPC collaboration exist for infections beyond SARS-CoV-2.^31^ A survey from Tomczyk et al^32^ highlighted the difficulties that the COVID-19 pandemic placed on antimicrobial resistance surveillance worldwide. More than half of the 73 countries of the WHO Antimicrobial Resistance (AMR) Surveillance and Quality Assessment Collaborating Centres Network surveyed reported decreased ability to work on AMR projects, decreased number of surveillance cultures, and decreased availability of nursing staff and reagents needed for laboratory testing.^32^ In the future, it will be even more important for ASP and IPC programs to work together to tackle AMR problems that have been exacerbated by the COVID-19 pandemic.

Prevention of infection is one of the best ways to decrease antimicrobial use, and vaccines are among the most powerful tools to accomplish this. Not only can vaccines protect an individual patient, and society at large, against infectious threats, vaccines can also be an important tool in society’s response to AMR.^33^ Nori et al^33^ argued that while ASP programs have not traditionally been involved in vaccine management, they are well positioned to assist in this process. ASP team members are frequently on pharmacy and therapeutic committees, have experience in the allocation of new and novel drugs, have pharmacy experts knowledgeable about drug transport and storage, have experience with coordinating with multiple different arms of the healthcare system, and are trusted leaders for information dissemination.^34^ Indeed, in a survey of 51 ASP leads, >90% reported that they were involved in the allocation of COVID-19 vaccines, which often required significant effort from the already strained ASPs.^16^ Further opportunities to improve vaccine uptake, including non–COVID-19 vaccines, are highlighted by the work of Shallal et al^35^ who demonstrated significant missed opportunities for vaccination against *Streptococcus pneumoniae* and influenza during the COVID-19 pandemic.

The COVID-19 pandemic has seen recognition of the remarkable work done by ID and critical care physicians, nurses, and other healthcare providers. However, we must acknowledge the specific challenges faced and the contributions of pharmacists to the pandemic response. Despite increased work responsibilities and work reassignments, pharmacists helped monitor COVID-19 medication use and adverse effects, helped manage drug shortages and supply chain issues, worked to preserve PPE, helped establish temporary COVID-19 hospitals during surges, wrote treatment guidelines, and provided education to clinicians and the public.^17,19,36^ Collins et al^37^ highlighted the importance of pharmacy care in their retrospective review of 197 patients with COVID-19 who had at least 1 pharmacist intervention during their hospital stay. These 197 patients had 15,818 medication days and received 1,572 pharmacist interventions (mean medication interventions per patient 8). Of these interventions, the most common were regimen simplification and medication timing to minimize the frequency of hospital staff room entry while caring for infected patients, followed by stewardship interventions to ensure optimal use of antimicrobials.^37^ More recently, with the authorization of new oral COVID-19 antiviral medications like ritonavir-boosted nirmatrelvir, pharmacists have again been of great importance in counseling, performing medication reconciliation, and mitigating potentially severe drug–drug interactions.^38^

## Impact of the COVID-19 pandemic on ASPs

Some authors have postulated that the increased focus on COVID-19 by ASPs could lead to decreased efforts towards non–COVID-19–related ASP targets, potentially leading to suboptimal antimicrobial prescribing.^39^ The surge in antibiotic utilization for COVID-19 patients and barriers to in-person interaction between ASPs and medical teams were additional challenges to successful stewardship.^16,17,40^ Vaughn et al^16^ distributed a survey to 51 ASP leads to assess how the role of ASPs changed during the pandemic. ASPs reported a median of 5 new duties and 82% reported an increase in work load. These additional duties included development of COVID-19 treatment guidelines, allocation of anti–COVID-19 medications, COVID-19 education to healthcare providers and the public, and isolation and PPE guidelines. Worryingly, only 8% of ASP leads reported increased full-time equivalent (FTE) support to deal with these new responsibilities and 18% reported decreased FTE. Also, 73% of respondents reported a decreased ability to perform normal stewardship activities and 71% had symptoms suggestive of burnout. Similarly, in a survey of 95 ASP leaders in the United Kingdom performed by Ashiru-Oredope et al,^17^ 64% of respondents reported that COVID-19 had a negative impact on ASP activities, including stewardship ward rounds, antimicrobial audits, and quality improvement interventions. Pandemic-related challenges have also decreased self-reported effectiveness of ASP teams, with respondents of one survey reporting reduced adherence to ASP recommendations, increased use and duration of broad spectrum antibiotics, and decreased screening for multidrug-resistant organisms.^40^

In response to patient-volume surges, hospitals frequently created new COVID-19 units to increase their critical-care capacity.^41^ These units were often staffed by internal providers outside their normal care area, such as pediatric providers pulled to take care of adult patients, or by traveling clinicians who were less familiar with institution-specific antibiograms, guidelines, and stewardship strategies.^15,42^ Consequently, compliance with existing stewardship practices may have decreased and suboptimal antimicrobial prescribing may have increased, although these trends have not been systematically studied. The sheer volume of critically ill and dying patients during the early phases of the pandemic may have also affected the success of antimicrobial stewardship interventions.^43^

Despite the numerous challenges, it is also important to acknowledge the potential benefits of the pandemic to ASPs. Published data suggest that the pandemic accelerated use of technology as part of some ASPs.^17^ Although the increased use of technology with stewardship comes with its own challenges, benefits such as increased efficiency for stewards and increased accessibility have been noted.^44^ Furthermore, work on high-visibility projects, such as vaccine response coordination, may help build relationships with hospital administration, while multidisciplinary work, for example on monoclonal antibody efforts, helps establish new and strengthen existing relationships with hospital colleagues. With this enhanced visibility, there is an opportunity for ASPs to highlight the impact of their work and credibly advocate for increased resources and protected time.

## Unique impacts on pediatric ASPs

Although much of the COVID-19 pandemic focus has been on adults, children have also been severely affected. The CDC estimates >1,200 pediatric deaths in the United States due to COVID-19 since the pandemic began.^45^ Pediatric health systems and ASPs have had to deal with similar challenges as adult providers, such as lack of effective treatment options early in the pandemic. However, pediatric ASPs faced several unique challenges.

As part of the pandemic response, many institutions and public health offices focused on adult care were able to hire dedicated staff or set up stand-alone infusion centers for COVID-19 monoclonal antibodies to manage the overwhelming patient volume.^46–48^ Despite the existence of several high-risk groups of children who might benefit from these therapies, the relatively low number of children compared to adults made hiring dedicated staff less practical and the burden of providing access to these medications often fell on pediatric ASPs.^49^

Early in the pandemic response, clinical trials identified a number of treatment modalities including remdesivir, dexamethasone, and COVID-19 monoclonal antibodies; unfortunately, children were excluded from these trials. This created several challenges. Although initial emergency use authorizations (EUAs) often included children 12 years of age and older, no pediatric data were available to guide the use of these investigational agents in adolescents, and pediatricians were forced to extrapolate potential risks and benefits of treatment from adult studies. Therefore, pediatric ASPs collaborated to evaluate risks and benefits of use of investigational drugs in adolescents and young children and created guideline documents.^50^ Children <12 years of age were an even greater challenge because they were not covered under initial EUAs, and pediatric ASPs were frequently called upon to help navigate the time-consuming process of obtaining compassionate use of these drugs through expanded access programs, which took time away from traditional ASP activities. Given the adult-oriented focus of the pandemic, initial supplies of COVID-19 monoclonal antibodies available to pediatric institutions were often limited, and ASPs were frequently called upon to help identify patients who might benefit the most from these treatments.

Like the aforementioned treatments, COVID-19 vaccines greatly influenced the management of the pandemic. Here again children were excluded from initial trials and approval. To date, >2 years into the pandemic, only 1 COVID-19 vaccine is available to children in the United States, and it is only authorized for children aged >5 years.^51^ Similarly to COVID-19 monoclonal antibodies, supplies of these vaccines were limited early in the pandemic, and the prioritization of higher risk adult patients forced ASPs to stratify pediatric patients risk based on limited data. In addition, pharmacists faced unique patient safety challenges with multiple vaccine formulations and doses approved for a single vaccine manufacturer, requiring additional logistical management to prevent vaccine dosing and administration errors in children.^52^

## Inclusion of ASPs as part of future pandemic responses

ASPs have been greatly impacted by the ongoing COVID-19 pandemic, but they have also been invaluable contributors to the pandemic response. Despite no mention of ASPs in previous pandemic guidance, we and others believe that ASPs should be part of future pandemic planning.^9,25,53^ The COVID-19 pandemic has proven just how quickly our existing healthcare systems can become overwhelmed by novel pathogens. As discussed earlier, ASPs have expertise, skill sets, and experience that can help assist hospital administrators, healthcare epidemiologists, and frontline healthcare personnel as they respond to these threats. To ensure that ASPs are able to meaningfully contribute, they must be integrated early on as part of the pandemic response. This inclusion will ensure clear lines of communication between ASPs and other key stakeholders and allows for early identification of additional resources that may be necessary to ensure success (eg, IT support, FTE allocation). For their part, ASPs are encouraged to think broadly and creatively about the scope of their work and engagement. For example, ASPs are encouraged to include all members of the healthcare system, as part of their work. Cortenay et al^54^ describe potential benefits of using nurses in ASP projects, including nurses’ frequent experience with collaboration and presence at bedside, which could help in distinguishing between viral and bacterial infections as well as identifying patients who may be appropriate for transition from IV to enteral antibiotic therapy. In addition, nurses’ frequent bedside contact is ideal for patient communication and engagement around the appropriate use of antimicrobials.^54^ Future collaboration between nursing and ASP may be beneficial, but programs will need to ensure that they are not placing increased burden on already overtaxed nurses. ASPs used their expertise in rational antimicrobial use to assist in allocation when anti–COVID-19 medications, such as remdesivir and COVID-19 monoclonal antibodies, were scarce. In the future, ASPs can consider extending these skills to vaccines and nontraditional antimicrobials such as convalescent plasma.^34,55^ Importantly, ASPs should prioritize the collection of data related to their pandemic response work so that those in charge of resourcing programs understand the breadth and depth of work that is happening. Disseminating generalizable knowledge to the medical community through publication is important as well.

In conclusion, ASPs around the world have stepped up to aid in the COVID-19 pandemic response. In addition to continuing their daily activities to protect patients from the adverse effects of antimicrobials and help slow the spread of antimicrobial resistance, ASPs have shaped many aspects of the pandemic response. ASPs worked to identify patients with COVID-19, and ensure they received appropriate treatment, all while helping health systems manage scare resources. There were both significant benefits to ASPs and significant costs of this pandemic-related work. In the future, ASPs should be considered part of a comprehensive pandemic response, but it will be important to ensure they are adequately resourced to do so.
